# Maternal perinatal social support and infant social-emotional problems and competencies: a longitudinal cross-cohort replication study

**DOI:** 10.1007/s00737-024-01473-x

**Published:** 2024-05-31

**Authors:** Melanie Schuijers, Christopher J. Greenwood, Jennifer E. McIntosh, George Youssef, Primrose Letcher, Jacqui A. Macdonald, Elizabeth Spry, Genevieve Le Bas, Samantha Teague, Ebony Biden, Elizabeth Elliott, Steve Allsop, Lucinda Burns, Craig A. Olsson, Delyse M. Hutchinson

**Affiliations:** 1 Centre for Social and Early Emotional Development, Deakin University, Burwood, Australia; 2 The Bouverie Centre, La Trobe University, Bundoora, Australia; 3 Murdoch Children’s Research Institute, Royal Children’s Hospital, Melbourne, Australia; 4 Department of Paediatrics, University of Melbourne, Melbourne, Australia; 5 Department of Psychology, College of Healthcare Sciences, James Cook University, Queensland, Australia; 6 Discipline of Child and Adolescent Health, University of Sydney, Sydney, Australia; 7 The Children’s Hospital at Westmead, Sydney, Australia; 8 National Drug Research Institute, Curtin University, Perth, Australia; 9 National Drug and Alcohol Research Centre, University New South Wales, Sydney, Australia

**Keywords:** Pregnancy, postpartum, social-emotional development, infancy, longitudinal

## Abstract

**Purpose:**

Maternal perinatal social support is theorised to promote offspring social-emotional development, yet few studies have prospectively examined this relationship. Findings may inform preventative intervention efforts, to support a healthy start to emotional life.

**Methods:**

This study examined whether maternal social support perinatally predicts infant social-emotional development at 12 months of age in two longitudinal cohort studies: The Australian Temperament Project (ATP) (*n* = 1,052 mother-infant dyads [653 mothers, *M*_age_at_birth_ = 32.03, 88% Australian-born; 1,052 infants, 52% girls]) and The Triple B Pregnancy Cohort Study (Triple B) (*n* = 1,537 dyads [1,498 mothers, *M*_age_at_birth_ = 32.53, 56% Australian-born; 1,537 infants, 49% girls]). Social support was assessed at pregnancy (third trimester) and eight-weeks post-birth. Infant social-emotional competencies (ATP: Brief Infant and Toddler Social and Emotional Assessment (BITSEA), Competencies Scale; Triple B: Bayley Scales of Infant and Toddler Development-Social Emotional Scale) and problems (ATP: BITSEA, Problems Scale; Triple B: Ages and Stages Questionnaires: Social-Emotional Scale), were assessed at 12-months of age.

**Results:**

In ATP, social support was associated with lower offspring problems (pregnancy: β = -0.15; post-birth: β = -0.12) and greater competencies (pregnancy: β = 0.12; post-birth: β = 0.16) at 12 months. In Triple B, social support also predicted lower offspring problems (pregnancy: β = -0.11; post-birth: β = -0.07) and greater competencies (pregnancy: β = 0.07) at 12 months. Findings did not indicate an association between support at eight-weeks post-birth and subsequent competencies (β = 0.06).

**Conclusions:**

Evidence suggests that perinatal social support promotes healthy infant social and emotional development. These results underscore the critical importance of social support for mothers transitioning into parenthood.

**Supplementary Information:**

The online version contains supplementary material available at 10.1007/s00737-024-01473-x.

The burden of mental disorders is significant, with a global prevalence rate of over 10% worldwide, and approximately 971 million people affected across all age groups (James et al. 2018). To reduce this burden, the World Health Organisation’s (WHO) Mental Health Action Plan 2013-2020 specified the implementation of strategies for prevention and promotion as major objectives. This has also been endorsed by the World Health Assembly in 2013 (World Health Organisation 2013). From a life course perspective, social-emotional problems during infancy can create and contribute to developmental vulnerabilities that increase risk for mental disorder across the life course (Bornstein 2014; Briggs-Gowan and Carter 2008). Conversely, social-emotional competencies established in infancy have been linked with mental wellness and protective pathways (Haapsamo et al. 2012). This makes investment in a healthy start to emotional life central to any long-term strategy for addressing the burden of mental disorder across the life course (Bolt et al. 2002).

Several risk and protective factors occurring during pregnancy and postpartum have been associated with offspring social-emotional development in infancy, yet high-quality evidence is lacking for many of them, particularly for contextual factors which remain under-researched (McIntosh et al. 2021). One potentially important contextual factor is maternal social support during the perinatal period. This refers to relational support, including practical and emotional assistance for a mother, during pregnancy and post-birth, with the perceived availability of support being particularly important (Leahy-Warren et al. 2018). Social support has been theorised to buffer stress during key transitional periods, such as the perinatal period (Leahy-Warren et al. 2018). During this period, heightened psychological distress is common among mothers, and has been linked to the transition from a familiar reality, to a novel and less certain one (Riecher-Rossler and Steiner 2005). A meta-analysis by O’Dea et al. (2023) of 133 studies indicated that 19% of mothers reported perinatal depression, and 13% reported anxiety (O’Dea et al. 2023). Importantly, psychological distress during the perinatal period has been associated with both mother-infant bonding problems, and poorer offspring social-emotional development (Le Bas et al. 2020, 2021, 2022; O’Dea et al. 2023; Rogers et al. 2020, 2023). Social support has been postulated to be an important modifiable factor that can buffer these risks (Taylor 2011), and could be strengthened through interventions within existing systems, such as hospital antenatal clinics, and during the postpartum period, via Maternal, Family, and Child Health services. Despite this potential, the intergenerational influence of perinatal social support remains under-researched.

Notably, few empirical studies have investigated the associations between maternal perinatal social support and infant social-emotional development specifically (McIntosh et al. 2021). There is also a paucity of longitudinal studies tracking distal outcomes of perinatal social support (i.e., beyond the early post-birth months), with inconsistent results among existing studies, and no replication studies published to date. The generalisability of findings has also been limited by small samples and a primary focus on high risk clinical and/or adolescent samples (Crnic et al. 1986; McDonald et al. 2016; Narvaez et al. 2013; Unger and Wandersman 1985).

The literature on social support has also typically been focussed on maternal outcomes or infant attachment status, with positive associations between social support and attachment security identified in a majority of studies (McIntosh et al. 2021). However, it is also important to study behavioural expressions of infant social-emotional development, including self-regulation, relations with others, and interactions with the environment, as they have been shown to predict psychological distress and mental health across the life course (Bornstein 2014). Furthermore, it is practical to assess early behavioural indicators and competencies both clinically and at the population level via parent-report measures (Pontoppidan et al. 2017).

The purpose of this exploratory study was to examine the associations between maternal social support in the third trimester of pregnancy and at eight-weeks post-birth and infant social-emotional development at 12-months of age. Specifically, the aims were threefold: 1) to study its association with infant competencies, 2) to study its association with infant problems, and 3) to replicate findings across two Australian longitudinal cohort studies during the perinatal period.

## Materials and methods

### Ethical considerations

Ethics approval was granted by relevant university, hospital and health services human research ethics committees.

### Design and participants

To increase confidence in results and inferences made, a cross-cohort replication design was used to investigate the association of perinatal maternal social support with infant social-emotional development. Data was from the Australian Temperament Project, Generation 3 Study (ATP) and the Triple B Pregnancy Cohort Study (Triple B), which had similar exposures and outcomes measured prospectively at the same time points. To summarise, maternal social support in pregnancy was assessed in the third trimester of pregnancy (between week 27 to the end of pregnancy), and social support postpartum was assessed at eight-weeks post-birth. Infant social-emotional competencies and problems were assessed at 12-months of age.

The ATP is an ongoing prospective study of parents and infants born to a 15-wave population-based cohort established in 1983. Generations 1 and 2 (*n* = 2,443) were recruited through community maternal and child health centres in 20 urban and 47 rural local government areas in the state of Victoria, Australia, when infants were 4-8 months old (Vassallo and Sanson 2013). The local government areas were randomly selected, on the advice of the Australian Bureau of Statistics, to provide a representative sample of Victoria. Across 2012-2018, Generation 2 participants who became parents were invited to participate in the Generation 3 study (703 parents with 1,167 offspring) (Olsson et al. 2022). Participants were provided a standardised description of the study and written consent was obtained, before being invited to complete a computer-assisted telephone interview or web survey. The current study includes only Generation 2 (mothers) and Generation 3 (their children) participants. Generation 1 relates to grandparents who are not included in this study.

Triple B is an Australian longitudinal study involving 1,534 pregnant women and their partners recruited across 2009 to 2013 from antenatal clinics at four major public hospitals in New South Wales and Western Australia (Hutchinson et al. 2018). A standardised script was used to describe the study to pregnant women and written informed consent was obtained. Computer-assisted telephone interviews were completed either in person, via phone, or web survey. To capture a diverse sample and ensure recruitment sites were proportionally represented, research assistants attended antenatal clinics at each hospital, across all days and months of the year.

For each cohort, we included mother-offspring dyads who provided maternal social support and/or infant social-emotional data (ATP: *n* = 1,052 dyads [653 mothers, 1,052 children]; Triple B: *n* = 1,537 dyads [1,498 mothers, 1,537 children]).

### Measures

#### Maternal social support

For ATP, the Maternity Social Support Scale (MSSS) was used to assess maternal social support in the third trimester and at eight-weeks post-birth via self-report (Webster et al. 2000). The MSSS comprises six items asking about support from friends, family, and partner. For example, “I have good friends who supports me”, and “My husband/wife/partner helps me a lot”. Responses were on a five-point Likert scale from *Never* to *Always*. For Triple B, social support was assessed somewhat differently at the two time-points. In trimester three of pregnancy, participants were asked to rate the following question on a five-point Likert scale, from *Very Unsatisfied* to *Very Satisfied*, “When you are having problems, are you satisfied with the support you get from friends?”. At eight-weeks post-birth, participants were asked to provide three scores on the helpfulness of support from friends, families, and partners on a four-point Likert scale from *Not Helpful* to *Very Helpful*, with these scores averaged to an overall social support score.

#### Infant social-emotional development

Infant social-emotional problems and competencies were assessed in both studies at 12-months of age via caregiver self-report instruments. We included measures of problems and competence as research suggests that they are not necessarily the inverse of each other and are predicted by different factors (McIntosh et al. 2021). For ATP, the Brief Infant Toddler Social Emotional Assessment (BITSEA) Problems and Competence scales were used (Briggs-Gowan 2004). The competence scale measured adaptive behaviours such as compliance, social skills, empathy, sustained attention, play skills, and mastery motivation. The problems scale measured problems such as externalising, internalising, and dysregulated behaviours. Items enquired about the frequency of behaviours on a three-point Likert scale from *Rarely* to *Always*. For Triple B, the Bayley Scales of Infant and Toddler Development, third edition, Social Emotional scale (Bayley-SE), assessed competence; and the Ages and Stages Questionnaire: Social-Emotional (ASQ:SE), assessed problems (Bayley 2006; Squires and Bricker 2009). The Bayley-SE assessed healthy social-emotional milestones based on age. Examples include self-regulation, interaction with the environment, emotional signalling, and social skills. Responses were on a six-point Likert scale from *None of the time* to *All of the time*. The ASQ:SE enquired about possible difficulties with emotion regulation, temperament, eating, sleeping, social interactions, and play on a three-point Likert scale from *Rarely or never* to *Most of the time*.

Further, to aid with the interpretation of scale scores, we derived binary variables for each scale using pre-specified cut-offs. For the BITSEA competency scale, scores less than or equal to 12 indicated a possible deficit/delay (Briggs-Gowan 2004), while for the problem scale, scores greater than or equal to 13 indicated possible problems. For the Bayley-SE, scores less than or equal to 6 were used to indicate possible challenges (Weiss et al. 2010), and for the ASQ-SE, scores greater than 48 indicated delays in social-emotional development (Squires et al. 2002).

#### Confounders

We selected confounding variables based on previous research suggesting associations between demographic or social factors and our exposures and outcomes of interest. Where such data were not collected in the relevant wave, factors assessed prior to the third trimester were included. Potential confounders in both cohorts were maternal age (years), education (<University vs ≥University), ethnicity (not Australia vs Australian) and financial status (ATP: ‘finding it very difficult to live comfortably’; Triple B: Socio-Economic Indexes for Areas [SEIFA] decile with greater numbers reflecting higher socio-economic context), and infant sex at birth (male vs female).

### Statistical analysis

The study used Stata version 17.0 for all analyses (StataCorp 2019). A series of linear regression analyses estimated the association between maternal social support in the third trimester and at eight-weeks post-birth, and infant social-emotional problems and competencies at 12-months for the ATP and Triple B cohorts. We employed a robust variance estimator to account for the clustering in both samples (i.e., multiple children per mother). Analyses were conducted separately for each cohort and for each exposure-outcome relationship, with models estimated both unadjusted and adjusted for potential confounding factors. Prior to analysis, exposure and outcome scores were transformed to z-scores so that standardised regression coefficients could be calculated. Standardised coefficients can be interpreted as the estimated differences in the outcome variables for every standard deviation change in the predictor variables. The study used established guidelines to interpret effect sizes, taking into account the cumulative influence of small effects over time and the context of a community rather than a clinical sample (Funder and Ozer 2019). Thus, 0.05 was considered very small, 0.10 small, 0.20 medium, 0.30 large, and greater than 0.40 very large.

Multiple imputation was used to handle missing data in the inferential analyses. Twenty complete datasets were imputed, based on a multivariate normal model (Lee and Carlin 2010). Binary variables were imputed as continuous variables and then back transformed with adaptive rounding following imputation (Bernaards et al. 2007). Estimates were obtained by pooling results across the 20 imputed datasets using Rubin’s rules (Rubin 1988).

## Results

Descriptive statistics for the ATP and Triple B samples are presented in Table 1. In regard to demographic characteristics, participants in the two cohorts were similar on mean maternal age (ATP=32.03 years; Triple B=32.53 years) and child sex (ATP=52% female; Triple B=49% female). In ATP, 88% of participants reported being Australian-born (12% were “born outside of Australia”), compared to 56% in Triple-B (44% “born outside of Australia”). In both cohorts, approximately two-thirds of participants had a university or higher education level (ATP=66%; Triple B=68%), and financial status scores above the mid-point on the respective scales in each cohort. Mothers in both cohorts also reported on average “adequate” levels of social support at each timepoint and, as expected for community samples, infant problems and competencies typically fell within the “healthy" range, with comparatively few infants being categorised as having potential problems.

**Table 1:** Descriptive statistics for unimputed ATP and Triple B data

	ATP (*N*=1052)					Triple B (*N*=1537)				
	N	M/n	SD/%	95% CI	% miss	N	M	SD	95% CI	% miss
Social support exposure										
3rd trimester pregnancy ^a^ (M/SD)	734	27.51	2.28	(27, 28%)	30%	1516	4.42	0.77	(4, 4%)	1%
8-weeks post-birth ^b^ (M/SD)	551	27.43	2.37	(27, 28%)	48%	1112	2.49	0.52	(2, 3%)	28%
Outcomes (12-months)										
Infant competencies ^c ^(M/SD)	931	16.12	2.76	(16, 16%)	12%	1247	11.20	3.03	(11, 11%)	19%
Cut-off (n/%)	931	100	11%	(9, 13%)		1247	33	3%	(2, 4%)	
Infant problems ^d ^(M/SD)	932	7.13	4.32	(7, 7%)	11%	1247	20.52	13.49	(20, 21%)	19%
Cut-off (n/%)	932	109	12%	(10, 14%)		1247	45	4%	(3, 5%)	
Potential Confounders										
Maternal age (M/SD)	1052	32.03	2.35	(32, 32%)	0%	1535	32.53	5.11	(32, 33%)	<1%
Financial status (M/SD)	930	3.11	0.88	(3, 3%)	12%	1534	7.96	2.23	(8, 8%)	<1%
Education					31%					<1%
<University (n/%)	721	242	34%	(30, 37%)		1532	495	32%	(30, 35%)	
≥University (n/%)	721	479	66%	(63, 70%)		1532	1037	68%	(65, 70%)	
Ethnicity					73%					<1%
Australian (n/%)	281	247	88%	(84, 91%)		1532	855	56%	(53, 58%)	
Not Australian (n/%)	281	34	12%	(9, 16%)		1532	677	44%	(42, 47%)	
Child sex					0%					3%
Male (n/%)	1052	510	48%	(45, 52%)		1496	770	51%	(49, 54%)	
Female (n/%)	1052	542	52%	(48, 55%)		1496	726	49%	(46, 51%)	

*ATP *Australian Temperament Project, *Triple B *Triple B Pregnancy Cohort Study, *SD* standard deviation, *N* available data for each variable; ^a^=Social support in 3rd trimester of pregnancy assessed with the MSSS (ATP) and single-item (Triple B); ^b^=Social support 8-weeks post-birth assessed with the MSSS (ATP) and helpful support items (Triple B); ^c^﻿=Infant competencies assessed with the BITSEA competencies (ATP) and BAYLEY-SE Competencies (Triple B), ^d^=Infant problems assessed with the BITSEA problems (ATP) and ASQ-SE problems (Triple B)

Table 2 presents the associations between maternal social support in the third trimester of pregnancy and at eight-weeks post-birth, and infant social-emotional problems and competencies at 12-months of age in the ATP and Triple B cohorts. Supplementary logistic regression analyses using pre-specified outcome cut-off scores supported the pattern of findings (see Table S1 of the Supplement).

**Table 2:** Linear regression estimates for the associations between maternal perinatal social support and infant social-emotional outcomes at 12-months

	ATP (*N*=1052)				Triple B (*N*=1537)			
	BITSEA Competencies		BITSEA Problems		BAYLEY-SE Competencies		ASQ-SE Problems	
	β (95% CI)	*p*	β (95% CI)	*p*	β (95% CI)	*p*	β (95% CI)	*p*
Social Support: Third Trimester of Pregnancy								
Unadjusted	.12 (.04, .21)	.003	-.17 (-.25, -.09)	<.001	.07 (.01, .13)	.029	-.12 (-.18, -.06)	<.001
Adjusted	.12 (.04, .19)	.003	-.15 (-.23, -.07)	<.001	.07 (.01, .13)	.022	-.11 (-.17, -.05)	<.001
Social Support: Eight-weeks Post-Birth								
Unadjusted	.18 (.10, .26)	<.001	-.14 (-.23, -.05)	.002	.05 (-.02, .12)	.178	-.07 (-.13, -.01)	.032
Adjusted	.16 (.08, .25)	<.001	-.12 (-.21, -.03)	.012	.06 (-.01, .13)	.119	-.07 (-.13, -.01)	.032

Mother age, education, ethnicity, financial status, and infant sex were included in the adjusted models for both the ATP and Triple B cohorts. *ATP *Australian Temperament Project, *Triple B *Triple B Pregnancy Cohort Study, *BITSEA* Brief Infant and Toddler Social Emotional Assessment, *ASQ:SE* Ages and Stages Questionnaires: Social Emotional, *BAYLEY-SE* Bayley Scales of Infant and Toddler Development, Social Emotional scale

In ATP, after accounting for potential confounding factors, maternal social support in the third trimester of pregnancy was associated with lower offspring problems at 12 months of age (β=-0.15, 95% CI = -0.23, -0.07). Maternal social support at eight-weeks post-birth was also associated with lower problems at 12 months (β=-0.12, 95% CI = -0.21, -0.03). Similarly, maternal social support in the third trimester of pregnancy was associated with greater offspring competencies at 12 months (β=0.12, 95% CI = 0.04, 0.19). An association was also observed between social support at eight-weeks post-birth and greater infant competencies at 12 months (β=0.16, 95% CI = 0.08, 0.25). Associations were of a small-medium strength and were of similar strength after accounting for potential confounding factors.

In Triple B, after accounting for potential confounding factors, maternal social support in the third trimester of pregnancy was associated with lower offspring problems at 12 months (β=-0.11, 95% CI = -0.17, -0.05). Maternal social support at eight-weeks post-birth was also associated with lower problems at 12 months (β=-0.07, 95% CI = -0.13, -0.01). Similarly, maternal social support during the third trimester of pregnancy was associated with greater subsequent offspring competencies at 12 months (β=0.07, 95% CI = 0.01, 0.13); however, evidence did not support associations at eight-weeks post-birth (β=0.06, 95% CI = -0.01, 0.13). Associations were of a very-small to medium strength and were of similar strength after prior to accounting for potential confounding factors.

## Discussion

The current study provides evidence via cross-cohort replication for an association between maternal perinatal social support and infant social-emotional development. Across cohorts and perinatal time-points, higher maternal social support was associated with fewer social-emotional problems, and more social-emotional competencies, in one-year-old offspring. The only exception to this was a lack of statistical association between social support at eight-weeks post-birth and infant competencies in Triple B, albeit the relationship was in the expected direction. Generally, we found very small to medium effect sizes, which represent modest yet consistent meaningful differences from a population level prevention perspective (Funder and Ozer 2019). Results were consistent regardless of adjustment, suggesting that these associations were not attenuated by measured demographic factors. The use of population rather than clinically indicated samples provides new information capable of informing population level preventive intervention. Inclusion of behavioural measures of infant social-emotional development, spanning both problems and competencies, also supports the importance of perinatal maternal social support in both risk and protective intergenerational pathways.

The mechanisms via which social support may influence infant social-emotional development are likely complex. Conceptually, social relationships have been theorised as promoters of mental health through the support they confer (Leahy-Warren et al. 2018). Perceived availability of social support may act to buffer stress, facilitating the major transitions of pre-pregnancy to pregnancy, and pregnancy to parenthood (Leahy-Warren et al. 2018). Meta-analytic evidence suggests that social support can influence factors related to infant social-emotional development, such as maternal mental health, attitudes towards offspring and the parent role, and behavioural responses to an infant’s physical and emotional needs (Andresen and Telleen 1992; Harandi et al. 2017). There is also evidence that social support is associated with physical development in utero and reduced risk of preterm birth, which is in turn linked to improved offspring social-emotional development (Cassiano et al. 2016; Mirabzadeh et al. 2013). It is likely that intergenerational social-emotional developmental pathways are multifactorial, with social support potentially influencing a number of biopsychosocial and contextual variables via the reduction of stress.

### Implications

Findings underscore the potential value of greater investment in social support for women during the third trimester of pregnancy and the early post-birth period. This aligns with WHO recommendations and clinical guidelines calling for both medical and emotional care for women perinatally (National Institute for Health and Care Excellence 2014; World Health Organisation 2018). Preventative intervention could occur broadly at the population level (e.g., through structured social support opportunities), or be targeted towards individuals identified as having low social support (e.g., through clinical intervention).

In addition, results provide support for extended assessment of social support in the perinatal period. This study also contributes to the broader endeavour of establishing population level screening for factors which may have intergenerational ramifications. Specifically, we contribute replicated evidence supporting the inclusion of maternal social support as a predictive domain for inclusion within prognostic perinatal screening instruments assessing infant social-emotional development.

Pertaining to both population level intervention and screening, facilitatory infrastructure already exists within some public health systems, such as hospitals and Maternal, Family, and Child Health services. In the Australian context, for example, the latter are public health services that typically offer parent groups commencing in the post-birth period. This research supports the extension of such opportunities to earlier in the perinatal period. Timely intervention with the goal of increasing support is also warranted. Current patterns of attendance at hospital and Maternal, Family, and Child Health Services appointments suggest an optimal window for screening and intervention from approximately 20-weeks’ gestation to four-months post-birth (The Victorian Auditor-General’s Office (VAGO) 2011). We suggest screening and intervention as early as feasible after 20-weeks’ gestation, to both increase social support early in pregnancy and promote the potentially protective role of social support.

### Limitations

In mature cohort studies there are important sources of selection, measurement, and confounding bias that require consideration (Sterne et al. 2016). Attrition and non-response in both cohorts (ATP: 20-30%; Triple B: 16%) may limit the generalisability of findings, although we used multiple imputation to account for missingness. While there were minimal differences in demographics between those who continued and discontinued participation (mothers who continued participation were more likely to have parents born in Australia in ATP, and more likely to be of higher socio-economic status in Triple B), these were accounted for in the analyses. Further, it is possible that results were affected by residual attrition bias, where a sample is advantaged compared to the general population due to decreased likelihood of ongoing participation in the face of difficulty. Moreover, as the samples studied represented a high-income, Western, industrialised population, inferences from our samples may only be generalisable to similar populations. Our findings may also only be generalisable to mothers, as we did not examine social support in partners. Future research investigating partner social support would be beneficial as the literature has been predominantly focused on mothers.

The measurement of maternal social support also varied between cohorts and timepoints. While measures in ATP were consistent across time and encompassed support across family, friends, and partners, Triple B captured support from family, friends, and partners at the eight-week assessment, but only from friends during the pregnancy assessment. Additionally, in Triple B, level of support could not be ascertained from one mother who reported having no available supports. Future studies on such high-risk women is warranted to better understand the relationship between no social support and maternal and child outcomes. Despite these measurement differences, findings were robust to study differences, with all observed effects in the expected direction (i.e., higher maternal social support was associated with more optimal development). Further, as the measures used in both studies were self-report, they could have been influenced by social desirability response bias, potentially attenuating the observed associations. However, measurement occurred in a context of confidentiality, which likely reduced bias (Newman et al. 2017).

Finally, although we controlled for various potential confounders, it is possible that results reflect residual effects of unmeasured or uncontrolled factors. We nevertheless considered findings meaningful in their predictive capacity at the community level. Similarly, although we cannot claim causality between perinatal maternal social support and offspring social-emotional outcomes, it can be assumed that the former predicts the latter.

## Conclusion

Maternal social support during late pregnancy and early in the post-birth period is associated with infant social-emotional trajectories. Given social support is modifiable, this is a highly accessible protective factor that could reduce offspring problems and increase competence. In the context of the extant literature, there is growing evidence to suggest that increased social support may be beneficial in promoting intergenerational social-emotional health and protecting against problems at both the community and individual levels. The present research further highlights that infants’ broader socio-ecological contexts are critical to their wellbeing.

## Supplementary information

ESM 1 (DOCX 16.8 kb)

## Data Availability

Group-level data may be made available on request from the authors.
