# Supplementary material for: Maternal perinatal social support and infant social-emotional problems and competencies: a longitudinal cross-cohort replication study
Source: Arch Womens Ment Health. 2024 May 31;27(6):1033–41. doi: 10.1007/s00737-024-01473-x (PMC11579112; doi:10.1007/s00737-024-01473-x)
Supplement: Supplementary file 1 — (DOCX 16.8 kb) [file 737_2024_1473_MOESM1_ESM.docx]

| Table S1  *Logistic regression estimates for the associations between maternal perinatal social support and infant social-emotional outcomes at 12-months.* | | | | | | | | |
| --- | --- | --- | --- | --- | --- | --- | --- | --- |
|  | ATP (*N* = 1052) | | | | Triple B  (N=1537) | | | |
|  | BITSEA  Competencies (possible deficit/delay) | | BITSEA  Problems (possible problems) | | BAYLEY-SE  Competencies  (possible challenges) | | ASQ-SE  Problems  (delays in SE development) | |
|  | β (95% CI) | *p* | β (95% CI) | *p* | β (95% CI) | *p* | β (95% CI) | *p* |
| **Social Support:  Third Trimester of Pregnancy** |  |  |  |  |  |  |  |  |
| Unadjusted | .83 (.67, 1.02) | .074 | .77 (.63, .95) | .015 | .67 (.49, .90) | .008 | .74 (.58, .95) | .016 |
| Adjusted | .83 (.67, 1.04) | .104 | .79 (.64, .98) | .035 | .68 (.51, .92) | .013 | .76 (.59, .98) | .037 |
| **Social Support:  Eight-weeks Post-Birth** |  |  |  |  |  |  |  |  |
| Unadjusted | .72 (.57, .91) | .006 | .76 (.61, .95) | .015 | 1.15 (.71, 1.85) | .575 | .78 (.58, 1.05) | .100 |
| Adjusted | .73 (.57, .94) | .013 | .79 (.62, 1.01) | .058 | 1.20 (.72, 2.00) | .484 | .78 (.57, 1.08) | .130 |
| Note. Mother age, education, ethnicity, financial status, and infant sex were included in the adjusted models for both the ATP and Triple B cohorts.  ATP = Australian Temperament Project. Triple B = Triple B Pregnancy Cohort Study. BITSEA = Brief Infant and Toddler Social Emotional Assessment.  ASQ:SE = Ages and Stages Questionnaires: Social Emotional. BAYLEY-SE = Bayley Scales of Infant and Toddler Development, Social Emotional scale. | | | | | | | | |
